# Revisiting the placental clock: Early corticotrophin-releasing hormone rise in recurrent preterm birth

**DOI:** 10.1371/journal.pone.0257422

**Published:** 2021-09-16

**Authors:** Christina L. Herrera, Maria E. Bowman, Donald D. McIntire, David B. Nelson, Roger Smith

**Affiliations:** 1 Department of Obstetrics & Gynecology, University of Texas Southwestern Medical Center, Dallas, Texas, United States of America; 2 Mothers and Babies Research Centre, Hunter Medical Research Institute, University of Newcastle, Newcastle, New South Wales, Australia; Poissy-Saint Germain Hospital/Versailles Saint Quentin University, FRANCE

## Abstract

**Objective:**

To determine if maternal plasma CRH and preterm birth history were associated with recurrent preterm birth risk in a high-risk cohort.

**Study design:**

Secondary analysis of pregnant women with a prior preterm birth ≤35 weeks receiving 17-alpha hydroxyprogesterone caproate for the prevention of recurrent spontaneous preterm birth. All women with a 24-week blood sample were included. Maternal plasma CRH level at 24- and 32-weeks’ gestation was measured using both enzyme-linked immunosorbent assay (ELISA) and extracted radioimmunoassay (RIA) technologies. The primary outcome was spontaneous preterm birth <37 weeks. The association of CRH, prior preterm birth history, and the two combined was assessed in relation to recurrent preterm birth risk.

**Results:**

Recurrent preterm birth in this cohort of 169 women was 24.9%. Comparing women who subsequently delivered <37 versus ≥37 weeks, mean levels of CRH measured by RIA were significantly different at 24 weeks (111.1±87.5 vs. 66.1±45.4 pg/mL, P = .002) and 32 weeks (440.9±275.6 vs. 280.2±214.5 pg/mL, P = .003). The area under the receiver operating curve (AUC) at 24 and 32 weeks for (1) CRH level was 0.68 (95% CI 0.59–0.78) and 0.70 (95% CI 0.59–0.81), (2) prior preterm birth history was 0.75 (95% CI 0.67–0.83) and 0.78 (95% CI 0.69–0.87), and (3) combined was 0.81 (95% CI 0.73–0.88, P = .001) and 0.81 (95% CI 0.72–0.90, P = .01) respectively for delivery <37 weeks. CRH measured by ELISA failed to correlate with gestational age or other clinical parameters.

**Conclusion:**

In women with a prior preterm birth, CRH levels were higher and had an earlier rise in women who experienced recurrent preterm birth. Second trimester CRH may be useful in identifying a sub-group of women with preterm birth due to early activation of the placenta-fetal adrenal axis. Assay methodology is a variable that contributes to difficulties in reproducibility of CRH levels in the obstetric literature.

## Introduction

Preterm birth occurred in 10.02% all of births in 2018 in the United States and remains a substantial cause of neonatal morbidity and mortality [1]. The etiology of preterm birth is multifactorial, resulting from inflammation from systemic disease or infection, abruption, uterine overdistention, abnormalities of the cervix and early activation of the maternal-fetal hypothalamic-pituitary adrenal systems [2]. Given the multifactorial pathogenesis and significant morbidity, there is a need to identify early markers of preterm birth to enable prediction and ultimately possible prevention strategies.

**Fig 1:**
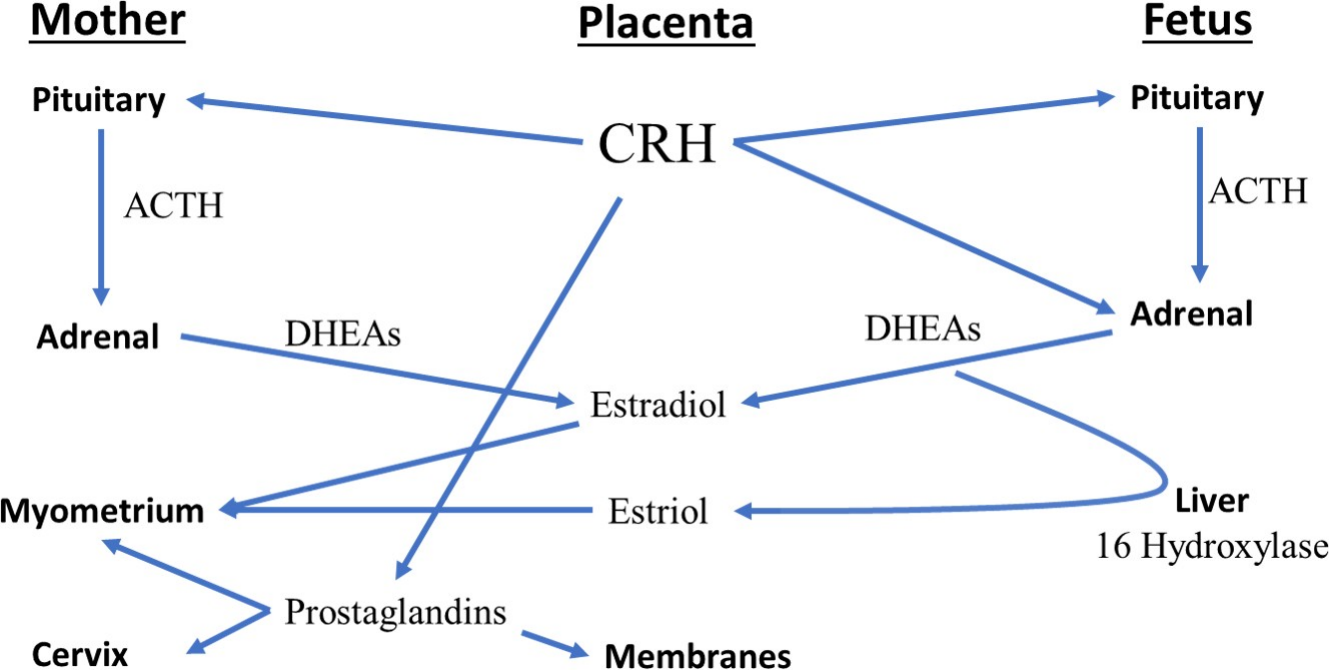
CRH pathways to labor. ACTH = adrenocorticotropic hormone, DHEAs = dehydroepiandrosterone sulfate.

Corticotropin-releasing hormone (CRH) has been investigated as a marker for preterm birth due to its endocrine, autocrine, and paracrine roles (Fig 1) [3]. Pregnancy is the only time when the peripheral circulation contains high concentrations of CRH, resulting from placental production [4]. Corticotropin-releasing hormone binding protein (CRH-BP) binds to CRH in the circulation reducing its activity until the binding protein becomes saturated late in pregnancy as CRH levels continue to rise [5]. CRH in pregnancy has been proposed to mediate its effects through its receptors in the placenta as a powerful vasodilator, in the myometrium potentiating signaling for either contractile or relaxatory mechanisms, and in the fetal adrenal as a stimulus for steroid production of dehydroepiandrosterone sulfate (DHEA-S), the precursor for placental production of estriol and estradiol [5]. CRH is suspected to contribute to preterm birth by increasing prostaglandins and estriol, which create an environment in favor of labor [6].

CRH was first suggested as a marker of a placental clock regulating the length of gestation in a seminal study by McLean et al. (1995) that found that measurement of CRH as early as 16–20 weeks identified women destined to deliver preterm as distinct from term or post-term delivery [7]. Multiple subsequent studies have confirmed an association between CRH and preterm birth; however, most are based on a single measurement of CRH in the second or third trimester in an unselected population with sensitivity and positive predictive values below 50% [3, 8–14]. Moreover, there are inconsistencies in the literature with other studies failing to observe an association between maternal plasma CRH concentrations and preterm birth [15, 16].

Women with a prior history of preterm birth have a subsequent risk of recurrent preterm birth that is in part determined by their prior history. Previous studies have shown that the number of preterm births, temporal order of prior preterm births, and severity (e.g. earlier gestational age) all interplay to determine a women’s subsequent preterm birth risk [17–19]. Based on these prior observations, we hypothesized that in women with a prior preterm birth history: (1) The earlier rise in CRH would distinguish women who would experience a recurrent preterm birth from those who would have a subsequent term birth, as has been variably reported in an unselected population; and (2) CRH levels, prior preterm birth history, and the combination of the two would be associated with recurrent preterm birth risk. We also compared CRH results obtained with an enzyme-linked immunosorbent assay (ELISA) with those using an extracted radioimmunoassay (RIA) given previous incongruous results.

## Materials and methods

### Study design

A prospective cohort study of women receiving 17-alpha hydroxyprogesterone caproate (17 OHP-C) was conducted and has previously been reported elsewhere [19]. Briefly, upon enrollment into prenatal care within the Parkland Health and Hospital System, women with a reported history of preterm birth were referred into a high-risk clinic for preterm birth. Criteria for referral included a current singleton gestation and prior spontaneous preterm birth or rupture of membranes between 20 0/7 and 35 0/7 weeks’ gestation. Women as part of this study underwent a detailed review of their obstetric history and a written informed consent process by a research nurse. The intent of the study was to introduce 17 OHP-C into the population and determine its effectiveness as therapy.

This is an analysis of a subset of patients enrolled in the parent study with maternal blood obtained from women delivering between August 2, 2014, and September 16, 2017. A total of 430 women with at least one prior spontaneous preterm birth between 20 0/7 and 35 0/7 weeks’ gestation were enrolled in the parent study and received weekly 250 mg intramuscular injections of 17 OHP-C commencing between 16 0/7 and 20 6/7 weeks’ and continuing until 36 6/7 weeks’ gestational age or delivery. These women were analyzed in a prospective inception cohort study that utilized a 3:1 matched historical cohort for preterm birth profile, maternal race, and body mass index (BMI). This trial found that 17 OHP-C was ineffective for prevention of recurrent preterm birth and was associated with an increased rate of gestational diabetes [19].

Venous blood was collected in a sub-cohort of the total study population coinciding with routine prenatal care blood draws at 24 and 32-weeks’ gestational age prior to administration of a scheduled weekly 17 OHP-C injection. Venous samples were collected from women who consented to blood collection and attended their prenatal visit during the gestational age window at 24 and 32 weeks. Samples obtained were processed within 4 hours of blood collection, aliquoted, and stored at -80°C until analyzed. This study was approved by the Institutional Review Board of the University of Texas Southwestern Medical Center and by the Human Research Ethics Committee of the University of Newcastle, Australia.

### CRH measurement

CRH levels were initially assessed via enzyme-linked immunosorbent assay (ELISA) kit (LifeSpan Biosciences, Seattle, WA) without extraction. Analysis was performed at the University of Texas Southwestern Medical Center in batch fashion at a single laboratory by a research associate masked to pregnancy outcomes. All samples were run parallel in duplicate. The intra- and interassay coefficients of variance (CV) were 6.1 and 5.3% respectively.

Frozen plasma samples were also transported on dry ice to the Mothers and Babies Research Centre, University of Newcastle, Australia and processed as previously described [20]. Pregnancy outcomes were blinded during analysis. Briefly, samples were extracted with methanol and CRH was measured in duplicate using a radioimmunoassay (RIA). Extraction recovery was 87%. No correction of the data for extraction recoveries was made. The limit of sensitivity was 3 pg/mL. The intra- and interassay coefficients of variance (CV) were 7.3 and 8.7%, respectively. Results were expressed in pg/mL.

### Statistical analysis

Demographic data and two-group comparisons of delivery outcome (gestational ages <37 versus ≥37 weeks or <35 versus ≥35 weeks) data were analyzed using the Pearson chi-square test for categorical data and the Student’s t-test for continuous measures. If the continuous measures were not judged as a normal distribution, then the Wilcoxon rank-sum test was used. Due to differences in rates of PTB between Hispanic and Black patients, a two-factor (ethnicity, delivery outcome) analysis of variance with interaction was performed based on CRH level.

In order to present CRH levels across gestational ages for the delivery outcome categories, the CRH values were log transformed and an analysis of variance (ANOVA) with interaction was conducted on the transformed CRH levels. Significance levels and estimates were drawn from these transformed data. The estimates were then back-transformed to the measured domain using an exponential transformation prior to plotting.

To determine the association of CRH, prior preterm birth history, and the combination of these two with delivery outcome, the areas under the receiver operating characteristic curves were estimated through logistic regression. Prior preterm birth history was determined using a rank-order pattern of up to the last three deliveries. This pattern was based on the number and order of prior term and preterm deliveries. Further details on this rank-order pattern are provide in supporting information (S1 File). Areas under the curve were estimated for the CRH alone, preterm birth history alone, and the combination of the two factors with 95% confidence intervals.

Given that all women in the cohort received weekly 17 OHP-C injections a Pearson correlation coefficient was calculated for 17 OHP or 17 OHP-C maternal blood levels and CRH at 24 weeks.

## Results

**Fig 2:**
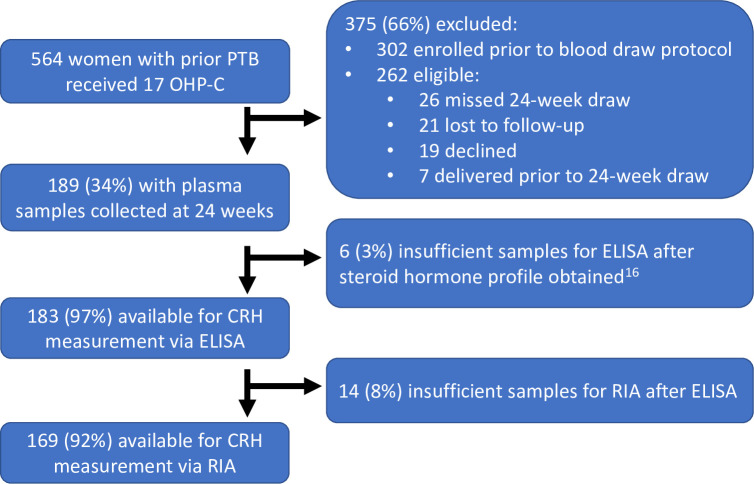
Study flow diagram. 17 OHP-C = 17-alpha hydroxyprogesterone caproate, ELISA = enzyme-linked immunosorbent assay, RIA = radioimmunoassay.

At our institution, 17 OHP-C was studied in a total of 564 women, and venous blood sampling was begun midway through the parent 17 OHP-C study (Fig 2). A total of 262 women were eligible for blood draw, and 243 consented for blood collection. A total of 189 samples were collected at 24 weeks, some of which underwent testing for their steroid-hormone profile [19]. Thus, of the original 262 eligible a total of 183 samples (70%) were ultimately available for the assessment of CRH by ELISA, and sufficient plasma for the subsequent CRH level measurement by extracted RIA was available for 169 participants (65%) at 24 weeks. A total of 113 patients had matched 32 weeks samples. Comparing women with and without samples, there was a higher rate of non-Hispanic black versus Hispanic women and a higher percentage of preterm birth in women without samples enrolled in the study (Table 1).

**Table 1:** Demographic characteristics and baseline risk factors between women with and without CRH measurements by radioimmunoassay (N = 262).

Baseline Characteristics	CRH Measurement		P-value^a^
	Yes	No	
	(N = 169)	(N = 93)	
**Maternal age (yrs)**	30.1±5.6	28.7±6.2	.099
**≥ 35**	37 (22%)	19 (20%)	.782
**Race/ethnicity**			.007
**Black**	16 (9%)	22 (24%)	
**White**	1 (1%)	1 (1%)	
**Hispanic**	152 (90%)	69 (74%)	
**Other**	0 (0%)	1 (1%)	
**Parity** ^**b**^			.700
**1**	51 (30%)	32 (34%)	
**2**	72 (43%)	35 (38%)	
**≥2**	46 (27%)	26 (28%)	
**BMI (kg/m** ^**2**^ **)**	33.4+5.9	34.2+6.9	.363
**Delivery GA (weeks)**			
**<37**	42 (25%)	37 (40%)	.012
**<35**	24 (14%)	28 (30%)	.002

Data reported as N (%) or mean ± standard deviation.

^a^P-value as per χ^2^ for categorical and Student’s t-test for continuous variables.

^b^Parity based on entry into pregnancy for CRH measurement. All women were at least P1.

The overall rate of recurrent preterm birth in the CRH measurement cohort of 169 patients was 24.9% at <37 weeks and 14.2% at <35 weeks. There were 15 indicated total births, only one of which was preterm at 35–36 weeks. All other preterm births were spontaneous. The population consisted of primarily young, obese, multiparous and predominantly Hispanic women, similar to the original cohort (Table 2). There were differences among race/ethnicity and body mass index between women who delivered less than 37 weeks and greater than or equal to 37 weeks. Differences in race/ethnicity and prior recurrent preterm birth history were also observed between women who delivered less than 35 weeks and greater than or equal to 35 weeks. Due to differences in rates of PTB between Hispanic and Black patients, a two-factor (race/ethnicity, delivery outcome) analysis of variance with interaction was performed and was not significant.

**Table 2:** Demographic characteristics and baseline risk factors of women with CRH measurements by radioimmunoassay (N = 169).

Baseline Characteristics	Delivery GA (weeks)		P-value^a^	Delivery GA (weeks)		P-value^a^
	<37	≥37		<35	≥35	
	(N = 42)	(N = 127)		(N = 24)	(N = 145)	
**Maternal age (yrs)**	29.6±6.3	30.0±5.4	.663	29.7±6.0	29.9±5.5	.827
**≥ 35**	11 (26%)	25 (20%)	.372	6 (25%)	30 (21%)	.633
**Race/ethnicity**			.008			< .001
**Black**	9 (21%)	7 (5%)		8 (33%)	8 (6%)	
**White**	-	1 (1%)		-	1 (1%)	
**Hispanic**	33 (79%)	119 (94%)		16 (67%)	136 (94%)	
**Parity** ^**b**^			.555	10 (42%)	43 (30%)	.478
**1**	16 (38%)	37 (29%)		8 (33%)	63 (43%)	
**2**	16 (38%)	55 (43%)		6 (25%)	39 (27%)	
**≥2**	10 (24%)	35 (28%)				
**BMI (kg/m** ^**2**^ **)**	31.5±6.3	33.7±5.9	.041	32.4±7.0	33.3±5.9	.521

Data reported as N (%) or mean ± standard deviation.

^a^P-value as per χ^2^ for categorical and Student’s t-test for continuous variables.

^b^Parity based on entry into pregnancy for CRH measurement. All women were at least P1.

**Fig 3:**
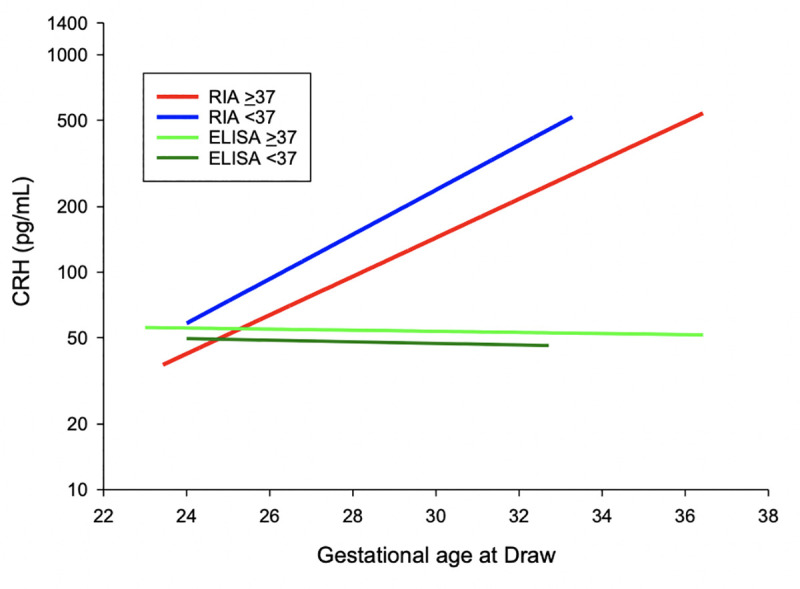
Initial CRH levels at 24 and 32 weeks by delivery outcome of <37 weeks as obtained by enzyme-linked immunosorbent assay (LifeSpan Biosciences) without extraction and by radioimmunoassay with extraction. Compared to radioimmunoassay with extraction, enzyme-linked immunosorbent assay without extraction results in assay binding interference and non-significant results. Enzyme-linked immunosorbent assay = ELISA, radioimmunoassay = RIA.

**Fig 4:**
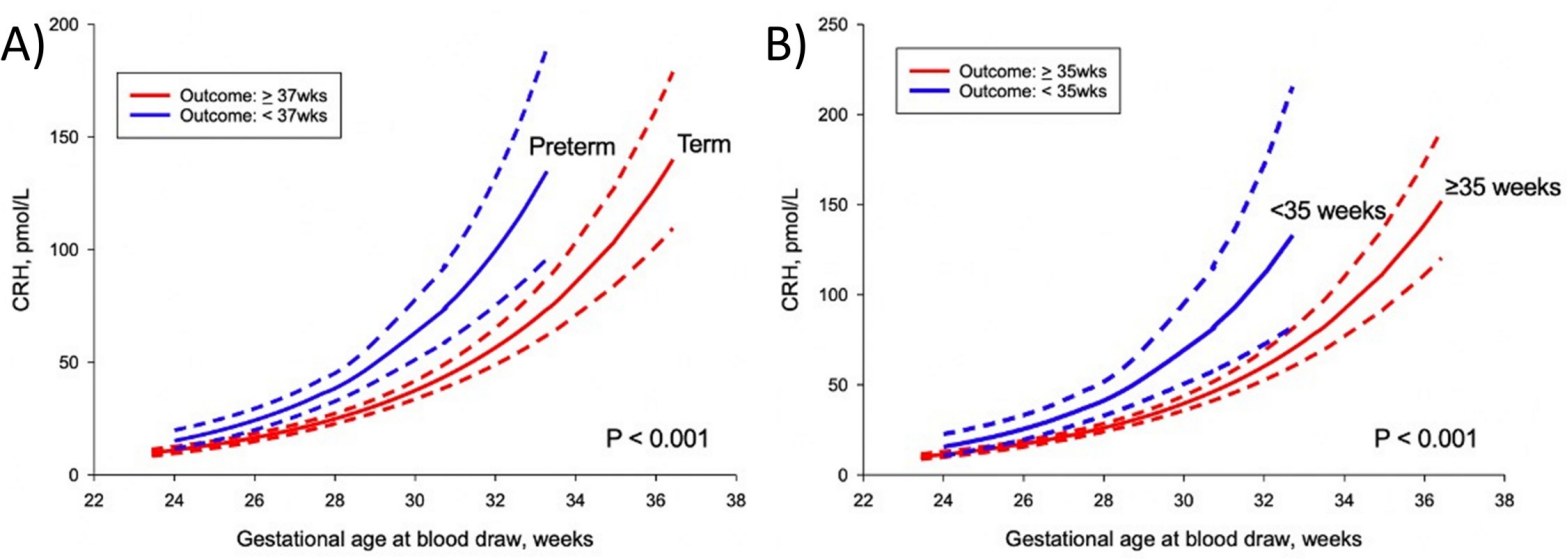
Maternal plasma corticotrophin-releasing hormone level as a function of gestational age based on delivery outcome: (A) <37 weeks (blue line) versus ≥37 weeks (red line); (B) <35 weeks (blue line) versus ≥35 weeks (red line). Dotted lines represent 95% confidence intervals.

Initial CRH results based on an ELISA for samples at 24 and 32 weeks showed a linear relationship without an increase across gestation (Fig 3). Due to these unanticipated results, CRH measurement was repeated via methanol extraction and then radioimmunoassay, Table 3 summarizes plasma CRH levels based on delivery outcome based on this method. Between women who experienced recurrent preterm birth versus a term delivery (<37 versus ≥37 weeks), mean levels of CRH were significantly different at 24 weeks (111.1±87.5 vs. 66.1±45.4 pg/mL, P = .002) and 32 weeks (440.9±275.6 vs. 280.2±214.5 pg/mL, P = .003). Between women who delivered <35 versus ≥35 weeks, mean levels of CRH at 24 weeks were also significantly different (126.9±105.5 vs. 69.1±46.7 pg/mL, P = .014). Median levels at these time points as well as the difference and rate of change between 32 and 24 weeks for delivery <37 weeks were also significantly different (Table 3). When measured by RIA CRH notably increased with increased gestational age and those with recurrent preterm delivery <37 weeks or <35 weeks have a significantly increased CRH compared to those delivering at term (Fig 4).

**Table 3:** Corticotrophin-releasing hormone level by delivery outcome (N = 169).

Gestational age, collection	Gestational age, delivery	N	Mean	P-value^a^	Median	P-value^a^
			(pg/mL)		(pg/mL)	
24 weeks	<37 weeks	42	111.1±87.5	.002	88.9 [49.3, 132.4]	< .001
	≥37 weeks	127	66.1±45.4		56.8 [33.6, 87.4]	
32 weeks	<37 weeks	24	440.9±275.6	.003	396.4 [248.3, 577.9]	.003
	≥37 weeks	89	280.2±214.5		235.5 [108.6, 371.7]	
Difference^b^	<37 weeks	24	334.3±202.3	.009	281.0 [198.7, 458.8]	.004
	≥37 weeks	89	218.9±183.5		158.6 [72.4, 290.0]	
Rate of change^c^	<37 weeks	24	61.9+51.4	.007	50.3 [28.1, 79.8]	< .001
	≥37 weeks	89	29.9+33.2		23.8 [11.6, 43.2]	
24 weeks	<35 weeks	24	126.9±105.5	.014	89.8 [50.6, 174.0]	.005
	≥35 weeks	145	69.1±46.7		58.0 [34.1, 91.4]	
32 weeks	<35 weeks	11	503.1±356.6	.083	426.0 [139.1, 779.2]	.037
	≥35 weeks	102	294.0±212.9		253.7 [114.1, 409.7]	
Difference^b^	<35 weeks	11	371.5±257.7	.103	337.0 [125.4, 544.2]	.054
	≥35 weeks	102	229.6±180.6		183.0 [77.6, 322.0]	
Rate of change^c^	<35 weeks	11	77.9+69.2	.055	56.2 [21.6, 105.3]	.012
	≥35 weeks	102	32.2+32.8		26.1 [11.9, 49.2]	

Data reported as mean ± standard deviation and median [1^st^ quartile, 3^rd^ quartile].

^a^P-value estimated using Student’s t-test and Wilcoxon rank-sum test, respectively.

^b^Difference represents the raw change between 32-week and 24-week paired samples.

^c^Rate of change represents the difference in paired samples over time (pg/mL/week).

A single CRH measurement at 24 and 32-weeks based on delivery outcome of less than 37 or 35 weeks was associated with an area under the receiver operating characteristic curve (AUC) of less than or equal to 0.70. Using the 24-week samples, the AUC was 0.68 (95% CI 0.59–0.78) for delivery <37 weeks and 0.68 (95%CI 0.55–0.84) for <35 weeks. For the 32 week samples, the AUC was 0.70 (95% CI 0.59–0.81) for delivery <37 weeks and 0.69 (95% CI 0.51–0.87) for <35 weeks. The minimum absolute difference of sensitivity and specificity was at a 24-week CRH level of 70 pg/mL (sensitivity 62%, specificity 63%) for delivery <37 weeks and 77 pg/mL (sensitivity 67%, specificity 66%) for <35 weeks. For the 32-week CRH level, these levels were 300 pg/mL (sensitivity 67%, specificity 64%) for deliveries <37 weeks and 310 pg/mL (sensitivity 64%, specificity 63%) <35 weeks respectively.

**Fig 5:**
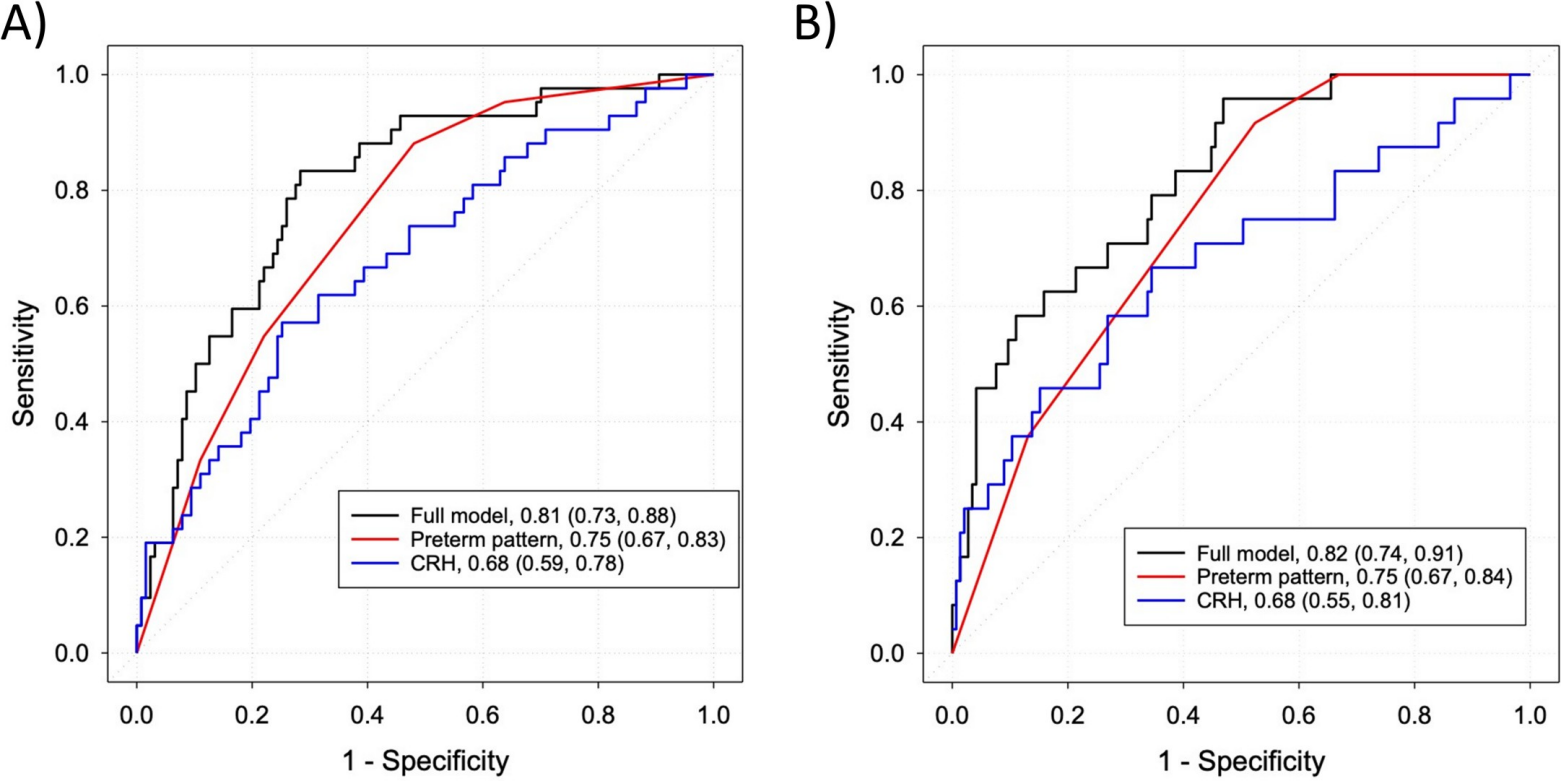
Receiver operator characteristic curves for plasma corticotrophin-releasing hormone levels and prior preterm birth history at 24 weeks based on delivery outcome of (A) <37 weeks and (B) <35 weeks. Green line = CRH alone. Red line = preterm birth history alone. Blue line = combination of CRH and prior preterm birth history. Area under the curve (AUC) statistics is 0.68 (95% CI 0.59–0.78) and 0.70 (95% CI 0.59–0.81) for CRH alone, at 24 (N = 169) and 32 (N = 113) weeks respectively, and 0.81 (95% CI 0.73–0.88, P = .001) for delivery <37 weeks and 0.82 (95% CI 0.74–0.91) for <35 weeks when CRH and prior preterm birth history are combined. The AUC combining CRH and prior preterm birth history is significantly different than that with CRH alone (P = .001 for <37 weeks and < .001 for <35 weeks).

Prior preterm birth history alone was slightly more strongly associated with recurrent preterm birth risk than CRH levels alone. Assessed at 24 weeks, the AUC was 0.75 (95% CI 0.67–0.83) for delivery < 37 weeks and 0.75 (95% CI 0.67–0.84) for delivery <35 weeks. Using 32 weeks, the AUC was 0.78 (95% CI 0.69–0.87) for delivery <37 weeks and 0.81 (95% CI 0.71–0.90) for delivery <35 weeks. The combination of CRH and prior preterm birth was significantly more associated with recurrent preterm birth risk than CRH alone (Fig 5, all P<0.03). Using the 24 week samples and prior preterm birth history, the AUC improved to 0.81 (95% CI 0.73–0.88, P = .001) for delivery <37 weeks and 0.82 (95% CI 0.74–0.91, P < .001) for <35 weeks. Using the 32-week samples, the AUC similarly improved to 0.81 (95% CI 0.72–0.90, P = .01) for delivery <37 weeks and 0.85 (95% CI 0.74–0.95, P = .03) for <35 weeks. There was no correlation found between 17 OHP or 17 OHP-C levels and CRH level at 24 weeks, with Pearson correlation coefficients of -0.059 and 0.121 (P = .51 and .18 respectively, S1 Fig).

## Discussion

### Principal findings

There were four principal findings for this study of CRH in women with prior preterm birth. Firstly, similar to women without a preterm birth history, in women with a prior spontaneous preterm birth CRH measured by extracted RIA increased with increasing gestational age. Secondly, those delivering either <37 weeks or <35 weeks had a significantly increased CRH compared to those delivering at term. Third, while CRH measurement alone had poor prognostic value, second trimester CRH in combination with prior preterm birth history was better able to identify a subgroup of women at risk for recurrent preterm birth. This indicates a potential ability to distinguish those whose preterm birth results from early activation of the placenta-fetal adrenal axis. Fourth, assay methodology is a variable that contributes to difficulties in reproducibility of CRH levels in the obstetric literature.

### Results

Our results are notable in that they confirm the rise in CRH is reproducible across variable populations, provided the correct methodology is followed. An earlier rise of CRH is observed in both women with and without a prior preterm birth history who experience preterm delivery in the observed pregnancy [3, 7–13]. Prior studies of unselected populations near our time points of interest and our measurements of CRH are show in Table 4. Compared to Inder et al. our levels are lower whereas for the remaining our levels at 24 and 32 weeks are the same or higher for a given gestational age. The variation in result is likely related to varying standards, methodology and study populations.

**Table 4:** CRH levels by RIA in unselected populations and in the current study.

Study	Gestational Age	CRH level (pg/mL)	
		Preterm	Term
	(Weeks)	(<37 weeks)	(≥37 weeks)
Wadhwa [3]	28–30	120.5±23.0	62.1±5.7
Hobel [9]	28–30	334.23±15.57	238.87±25.52
Inder [12]	26	466.7±81.8	165.1±11.4
Wadhwa [13]	33	215.0±31.5	139.6±11.7
Current study	24	111.1±87.5	66.1±45.4
	32	440.9±275.6	280.2±214.5

### Methodology considerations

Over the years there have been differences reported in plasma processing, extraction technique, as well as in the method of measurement of CRH [21]. Extraction is a step thought to be necessary as CRH is often heavily bound by plasma proteins, including by its named binding protein (CRH-BP) and the degree of this binding is highly variable between individuals [22]. Of the extraction techniques, methanol is thought to yield the best recovery [22]. Measurement is most commonly made by radioimmunoassay (RIA) [3, 7, 10, 13, 14, 20], but has also been obtained via enzyme-linked immunosorbent assay (ELISA) [12, 15].

There are two studies that used an ELISA (Pennisula Laboratories, San Carlos, CA) and performed no extraction technique. The findings of Moawad et al. (2002) reported at 24 weeks’ mean CRH levels of 0.3±0.1 mg/mL (3±1 x 10^8^ pg/mL) for cases who delivered <35 weeks and controls who delivered >37 weeks [12]. The other study by Siabi et al. (2005) looked at women with prior recurrent preterm birth and did not find a difference in levels of CRH obtained at 16–20 weeks in predicting delivery less than either 37 or 35 weeks [15]. They reported median levels of 390 pg/mL for <37 weeks and 370 pg/mL for ≥37 weeks (P = 0.08) and levels of 360 pg/mL for <35 weeks and 380 pg/mL for ≥35 weeks [15]. Additionally, they found no difference in measurements based on whether the woman was receiving 17-OHP-C treatment or placebo.

We speculate that the differences in our results stem from differences in methodology. When using an ELISA without an extraction step, our results were also non-significant in contrast to the RIA, likely from binding protein interference (Fig 4) [21]. It is for this reason we propose that future assessment of CRH should not be undertaken without a validated extraction technique as this appears to drastically affect results. Moreover, given the reproducibility of results demonstrated via RIA in this study as well as many others, this should be the preferred method of measurement in future research.

### Population considerations

Direct comparison of our results with others in the literature remains difficult due to variation among study populations. The only other study employing the exact same methods was in a predominantly White, unselected population without selection for prior preterm birth. The authors found 26-week median plasma CRH levels at 90.8 pg/mL in the term versus 115.2pg/mL in the preterm group (<37 weeks) [20]. Within our predominantly Hispanic population with a prior history of preterm birth, our mean levels at 24 weeks were 56.8 pg/mL for term deliveries and higher at 88.9 pg/mL for recurrent preterm birth. This suggests an earlier activation of CRH with recurrent preterm birth history.

We did note a higher rate of recurrent preterm birth in Black women in our population, though this finding is not wholly unexpected given that Black race is an independent risk factor for preterm birth [23]. Due to significant difference between PTB and race/ethnicity, we did assess for an interaction of race/ethnicity and preterm birth history on CRH and it was not significant. Overall, our study found a slightly higher sensitivity with a single measurement of CRH than previously reported. From our ROC curves the minimal difference of sensitivity and specificity for CRH alone was found to be at a sensitivity of 62–67%. The combination of CRH and prior preterm birth history did improve the association with delivery at <37 and <35 weeks. As our population was predominantly Hispanic, this finding warrants further validation in other populations.

### Research implications

We observed an early rise in CRH in women who experienced recurrent preterm birth compared to those who did not. From this observation, we hypothesize that CRH may well be a useful marker for identification of a subset of patients with early placental-fetal adrenal axis activation. Indeed, recent studies have supported the conceptual idea of a biologic clock accounting for differential biochemical reaction speeds [24–27]. In this light, the early synthesis and release of CRH by the placenta may well reflect early activation of the placental-fetal adrenal axis. Thus, CRH may be able to identify a specific subset of women at risk for preterm birth from the accelerated activation. Moreover, the interplay between prior preterm birth history and CRH level we see in our receiver operator characteristic curves (Fig 3) may in fact reflect this relationship. A larger, prospective study should be performed to confirm our observations. If confirmed, the improved ability to identify those at risk of preterm birth due to early activation of the placental-fetal adrenal axis could enable targeted intervention that is currently lacking.

Our data do not address a variety of other etiologies of preterm birth related to inflammation, abruption, cervical factors or uterine distention. Moreover, we recognize that other potentially useful predictive analytes and imaging were not obtained for this study, such as fetal fibronectin and cervical length [12, 28]. Cervical length specifically might have particular relevance to CRH, given that CRH does play a role in uterine prostaglandin production and could impact cervical length [6]. Further investigation to determine the appropriate combined analytes and imaging is also necessary to more accurately determine the risk for preterm birth due to different etiologies.

### Strengths and limitations

Strengths of this study include the reproducible approach taken for the measurement of CRH. Measurements were undertaken blinded to clinical outcome, and our results using RIA are remarkably similar to that of the original McLean paper despite a different population. The technique in measurement of CRH clearly is critical for the determination of levels. There are some limitations. Demographic characteristics may contribute to variation in CRH levels. Prior studies have suggested racial and ethnic variation in CRH levels. Based on studies by Holzman et al. (2001) and Siler-Khodr (2003), there is evidence that CRH levels in Black and Hispanic women are lower than White women [11, 29]. Yet, even so the trend for increasing CRH with recurrent preterm birth is still observed in our population.

Other limitations include that the results are based on clinical blood draws done closest to the target of 24 and 32 weeks. The 24-week samples were collected at a mean of 25.2±1.2 weeks and median of 25.0 [24.4,25.7] weeks. The 32-week samples were collected at a mean of 31.6±1.0 weeks and median of 31.6 [31.1,32.2] weeks. Plasma samples were not available from all participants, and it is possible there is selection bias among the samples tested. We did compare demographic data among participants with and without samples and found a greater percentage of non-Hispanic black compared to Hispanic women and a higher percentage of preterm birth in the unsampled women. The effect of 17 OHP-C injections on CRH levels is unclear. The only study to look for such an effect did not find a difference between women receiving 17 OHP-C and those that did not, but we have concerns with the methodology used in that study as outlined in the previous section [15]. We did compare levels of 17 OHP and 17 OHP-C with levels of CRH at 24 weeks’ and found no correlation. As we currently lack CRH data in women with preterm birth who have not received 17 OHP-C, we cannot make definitive conclusions on the impact of 17 OHP-C on CRH levels. Finally, there is also the possibility of sample degradation given the time from sampling to testing, as the oldest samples obtained were from 2014, but this would be expected to increase error in the assay and reduce the power of the study.

## Conclusions

In pregnant women with a prior history of spontaneous preterm birth higher maternal plasma CRH levels were observed with an earlier rise in those who experienced preterm birth. This is a similar to the rise of CRH in women without a history of preterm birth. The rise in maternal plasma CRH may be useful in identifying early activation of the placenta-fetal adrenal axis as an etiological factor in women at-risk for recurrent preterm birth. Assay methodology is a variable that contributes to difficulties in reproducibility in the obstetric literature.

## Supporting information

S1 Fig Scatterplot of 17-hydroxyprogesterone (17 OHP), 17-hydroxyprogesterone caproate(17 OHP-C) and CRH levels at 24 weeks. Units for 17 OHP ng/mL, 17 OHP-C ng/mL, and CRH pg/mL.
(TIF)

S1 File ROC curve construction. Explanation of rank-order applied for preterm birth history to construct ROC curve.
(DOCX)

S2 File Revised data analysis. Data Analysis by gestational age epochs and spontaneous preterm births.
(DOCX)

S3 File The shared dataset in SAS format. (SAS7BDAT)

S4 File An example program to read Shareddata.sas7bdat. This routine contains the formats applied to the variables in Shareddata.sas7bdat.
(SAS)

S5 File Data dictionary for the shared dataset. The data dictionary for the data in Shareddata.sas7bdat.
(DOCX)
