# Supplementary figures and images for: Revisiting the placental clock: Early corticotrophin-releasing hormone rise in recurrent preterm birth
Source: PLoS One. 2021 Sep 16;16(9):e0257422. doi: 10.1371/journal.pone.0257422 (PMC8445461; doi:10.1371/journal.pone.0257422)

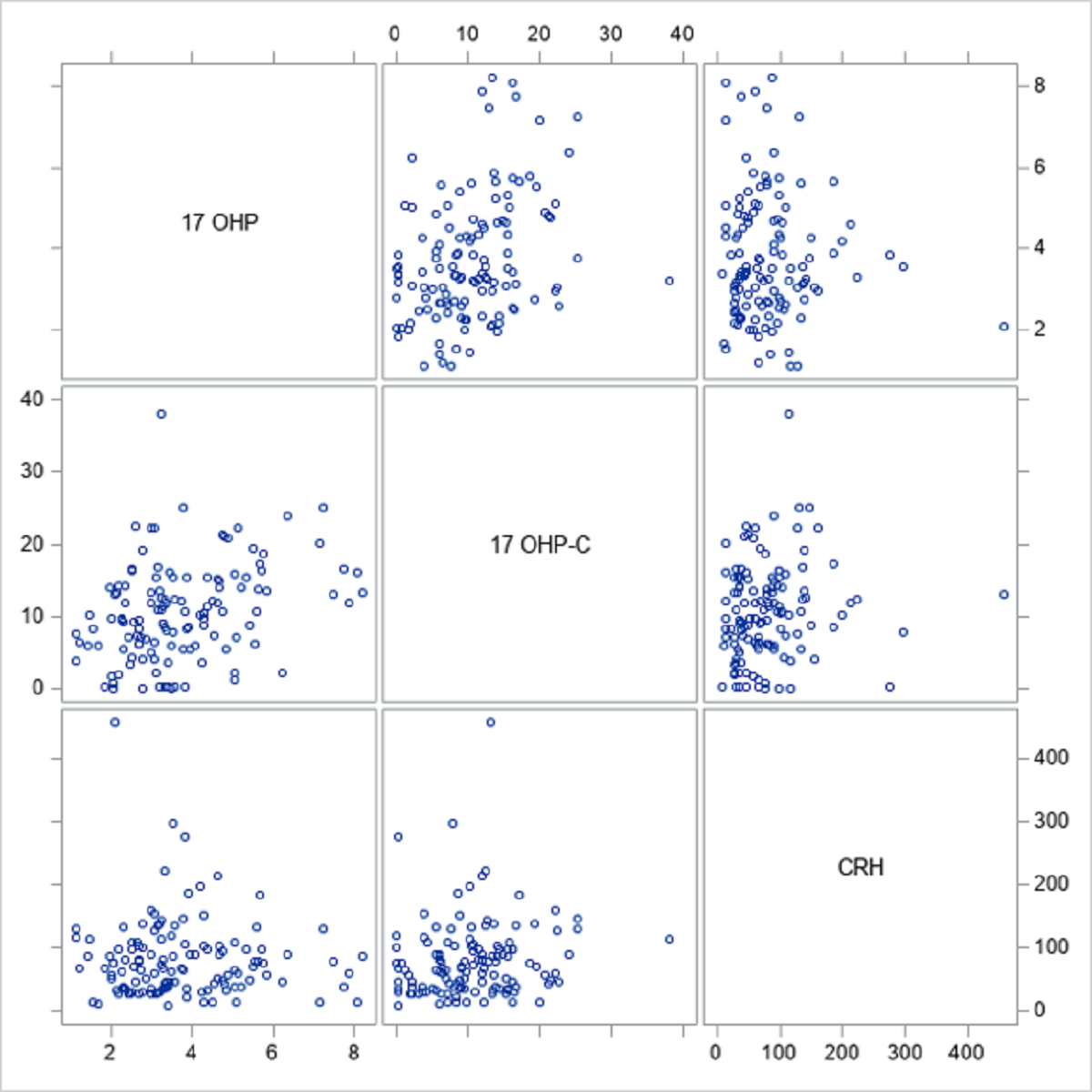

Supplement: S1 Fig — Units for 17 OHP ng/mL, 17 OHP-C ng/mL, and CRH pg/mL. (TIF) [file pone.0257422.s001.tif]
