# Supplementary material for: Revisiting the placental clock: Early corticotrophin-releasing hormone rise in recurrent preterm birth
Source: PLoS One. 2021 Sep 16;16(9):e0257422. doi: 10.1371/journal.pone.0257422 (PMC8445461; doi:10.1371/journal.pone.0257422)
Supplement: S1 File — Explanation of rank-order applied for preterm birth history to construct ROC curve. (DOCX) [file pone.0257422.s002.docx]

**Supporting file 1: ROC curve construction**

Preterm birth pattern is taken as follows in order of increasing risk of subsequent preterm delivery.

| Pattern of at most last three deliveries | Order |
| --- | --- |
| PPP | 1 |
| PP | 2 |
| Last three with Preterm in last delivery (e.g. PTP, TPP) | 3 |
| P | 4 |
| TP | 5 |
| PT | 6 |
| Other last three, T in last delivery (e.g. TPT, PTT) | 7 |

P = preterm, T = term

ROC curves were then constructed using CRH level at 24 weeks alone, the pattern of the last three deliveries (prior preterm birth history) alone, and then the two combined to generate Figure 4.
