# Supplementary material for: Revisiting the placental clock: Early corticotrophin-releasing hormone rise in recurrent preterm birth
Source: PLoS One. 2021 Sep 16;16(9):e0257422. doi: 10.1371/journal.pone.0257422 (PMC8445461; doi:10.1371/journal.pone.0257422)
Supplement: S2 File — Data Analysis by gestational age epochs and spontaneous preterm births. (DOCX) [file pone.0257422.s003.docx]

**Supporting File 2: Revised data analysis**

**Table 2: Demographics as modified by gestational age epochs**

| Characteristic | <35 | 35-36 | >=37 | P-value |
| --- | --- | --- | --- | --- |
| n | 24 | 18 | 127 |  |
| Age |  |  |  |  |
| mean+std dev | 29.7+6.0 | 29.4+6.8 | 30.0+5.4 | 0.902 |
| >=35 | 6 (25%) | 5 (28%) | 25 (20%) | 0.656 |
| Race/ethnicity |  |  |  | <0.001 |
| Black | 8 (33%) | 1 (6%) | 7 (6%) |  |
| White | 0 (0%) | 0 (0%) | 1 (1%) |  |
| Hispanic | 16 (67%) | 17 (94%) | 119 (94%) |  |
| Parity |  |  |  | 0.784 |
| 1 | 10 (42%) | 6 (33%) | 37 (29%) |  |
| 2 | 8 (33%) | 8 (44%) | 55 (43%) |  |
| >2 | 6 (25%) | 4 (22%) | 35 (28%) |  |
| BMI |  |  |  |  |
| mean+std dev | 32.4+7.0 | 30.2+5.1 | 33.7+5.9 | 0.065 |

**Table 3: CRH levels as modified by gestational age epochs**

| GA collection | GA at del | n | mean+std | P-value | med [Q1. Q3] | P-value |
| --- | --- | --- | --- | --- | --- | --- |
| 24 wks | <35 | 24 | 126.9+105.5 | 0.001 | 28.8 [50.6, 174.0] | 0.002 |
|  | 35-<37 | 18 | 90.2+51.0 |  | 87.4 [49.3, 101.8] |  |
|  | >=37 | 127 | 66.1+45.4 |  | 56.8 [33.6, 87.4] |  |
| 32 wks | <35 | 11 | 503.1+356.6 | 0.006 | 426.0 [139.1, 779.2] | 0.012 |
|  | 35-<37 | 13 | 388.3+181.8 |  | 395.7 [263.7, 540.3] |  |
|  | >=37 | 89 | 280.2+214.5 |  | 235.5 [108.6, 371.7] |  |
| Difference | <35 | 11 | 371.5+257.7 | 0.022 | 337.0 [125.4, 544.2] | 0.015 |
|  | 35-<37 | 13 | 302.8+144.2 |  | 263.3 [217.6, 433.0] |  |
|  | >=37 | 89 | 218.9+183.5 |  | 158.6 [72.4, 290.0] |  |
| Rate of change | <35 | 11 | 77.9+69.2 | 0.001 | 56.2 [21.6, 105.3] | 0.002 |
|  | 35-<37 | 13 | 48.3+25.2 |  | 49.8 [31.1, 72.2] |  |
|  | >=37 | 89 | 29.9+33.2 |  | 23.8 [11.6, 43.2] |  |

**Table 2: Demographics of spontaneous births by gestational age epochs.**

| Characteristic | <35 | 35-<37 | >=37 | P-value |
| --- | --- | --- | --- | --- |
| n | 24 | 17 | 113 |  |
| Age |  |  |  |  |
| mean+std dev | 29.7+6.0 | 29.1+6.8 | 29.8+5.3 | 0.863 |
| >=35 | 6 (25%) | 4 (24%) | 22 (19%) | 0.796 |
| Race/ethnicity |  |  |  | <0.001 |
| Black | 8 (33%) | 1 (6%) | 5 (4%) |  |
| White | 0 (0%) | 0 (0%) | 1 (1%) |  |
| Hispanic | 16 (67%) | 16 (94%) | 107 (95%) |  |
| Parity |  |  |  | 0.708 |
| 1 | 10 (42%) | 5 (29%) | 31 (27%) |  |
| 2 | 8 (33%) | 8 (47%) | 50 (44%) |  |
| >2 | 6 (25%) | 4 (24%) | 32 (28%) |  |
| BMI |  |  |  |  |
| mean+std dev | 32.4+7.0 | 30.5+5.2 | 33.2+5.7 | 0.209 |

**Table 3: CRH levels of spontaneous births as modified by gestational age epochs.**

| GA collection | GA at del | n | mean+std | P-value | med [Q1. Q3] | P-value |
| --- | --- | --- | --- | --- | --- | --- |
| 24 wks | <35 | 24 | 126.9+105.5 | 0.001 | 89.8 [50.6, 174.0] | 0.002 |
|  | 35-<37 | 17 | 91.4+52.3 |  | 87.4 [49.3, 101.8] |  |
|  | >=37 | 113 | 66.3+45.4 |  | 56.9 [33.6, 85.6] |  |
| 32 wks | <35 | 11 | 503.1+356.6 | 0.004 | 426.0 [139.1, 779.2] | 0.008 |
|  | 35-<37 | 13 | 388.3+181.8 |  | 395.7 [263.7, 540.3] |  |
|  | >=37 | 78 | 271.1+212.5 |  | 213.3 [108.6, 335.1] |  |
| Difference | <35 | 11 | 371.5+257.7 | 0.015 | 337.0 [125.4, 544.2] | 0.010 |
|  | 35-<37 | 13 | 302.8+144.2 |  | 263.3 [217.6, 433.0] |  |
|  | >=37 | 78 | 211.2+182.0 |  | 145.2 [66.1, 278.2] |  |
| Rate of change | <35 | 11 | 77.9+69.2 | 0.001 | 56.2 [21.6, 105.3] | 0.001 |
|  | 35-<37 | 13 | 48.3+25.2 |  | 49.8 [31.1, 72.2] |  |
|  | >=37 | 78 | 28.3+33.8 |  | 22.3 [11.1, 41.1] |  |
