# Supplementary material for: Revisiting the placental clock: Early corticotrophin-releasing hormone rise in recurrent preterm birth
Source: PLoS One. 2021 Sep 16;16(9):e0257422. doi: 10.1371/journal.pone.0257422 (PMC8445461; doi:10.1371/journal.pone.0257422)
Supplement: S5 File — The data dictionary for the data in Shareddata.sas7bdat. (DOCX) [file pone.0257422.s006.docx]

**Supporting File 5: Data dictionary for the shared dataset**

List of variables:

| Variable name | Label* | Description |
| --- | --- | --- |
| CRH24Ave | CRH at 24 weeks | CHR level at 24 weeks’ gestation |
| CRH32Ave | CRH at 32 weeks | CHR level at 32 weeks’ gestation |
| EGAatDelivery | Gestational age at delivery | Gestational age as the obstetrical age at delivery for which the delivery was managed. This includes ultrasound modification if it was deemed appropriate. This is completed weeks, meaning that a gestational age of 38 weeks and 2 days is recorded as 38 weeks. Similarly 38 weeks and 0 days is still 38 weeks. |
| Age | Maternal age at delivery | Maternal age in years at the time of delivery |
| Race | Maternal race | Maternal race. This is coded as 1=Black, 2=White, 3=Hispanic, 4=Asian (not occurring in this dataset), and 5=other (not occurring in this dataset). |
| BMI | Body mass index (kg/m2) at delivery | Body mass index (kg/m^2^) at delivery. This is based upon the maternal weight at delivery. |
| Parity | Parity prior to delivery (3=>2) | Parity prior to delivery (1 as para 1, 2 as para 2, and 3 as para >2). This is the parity during pregnancy and doesn’t include the current birth outcome. Para 0 is not possible in this dataset as inclusion on the parent study of this secondary analysis is at least one prior preterm delivery. |
| Number | Number of prior preterm births | Number of prior preterm births in the mother’s pregnancy history. Deliveries are considered only if gestational age is at least 19 weeks. By preterm in this definition it is meant that the delivery was < 37 weeks or < 37 weeks and 0 days. |
| PTB_Risk | Preterm birth risk category (based on prior deliveries) | Preterm birth risk category (based on prior deliveries). For patients with only one prior delivery if it was preterm then the value of PTB_Risk is 1. For two prior deliveries, then patients with both being preterm is coded 2, those with only one preterm delivery and it being the most recent then it is coded as 3, those with only on preterm delivery and it being the first is coded as a 4. For patients with three prior deliveries and all being preterm is coded as 5. For those with more than two prior deliveries and the last delivery is preterm (excluding those coded 5) then they are coded 6. All others with more than two prior deliveries are coded 7. Recall this study is a secondary analysis of a study where all patients had at least one prior preterm delivery, so there are no patients with no prior preterm deliveries. That’s why the coding for two prior deliveries does not have a category for both being term deliveries. |
| Patient ID | Patient ID | Unique integer for the patient identifier |

*The label is a SAS characteristic that is part of the data element as is the Variable name. The description is only available in this table.
